# Supplementary material for: Astaxanthin, a Marine Carotenoid, Maintains the Tolerance and Integrity of Adipose Tissue and Contributes to Its Healthy Functions
Source: Nutrients. 2021 Dec 6;13(12):4374. doi: 10.3390/nu13124374 (PMC8703397; doi:10.3390/nu13124374)
Supplement: Supplementary file 1 [file nutrients-13-04374-s001.zip › nutrients-1489680-supplementary.pdf]

Supplementary Figure S1

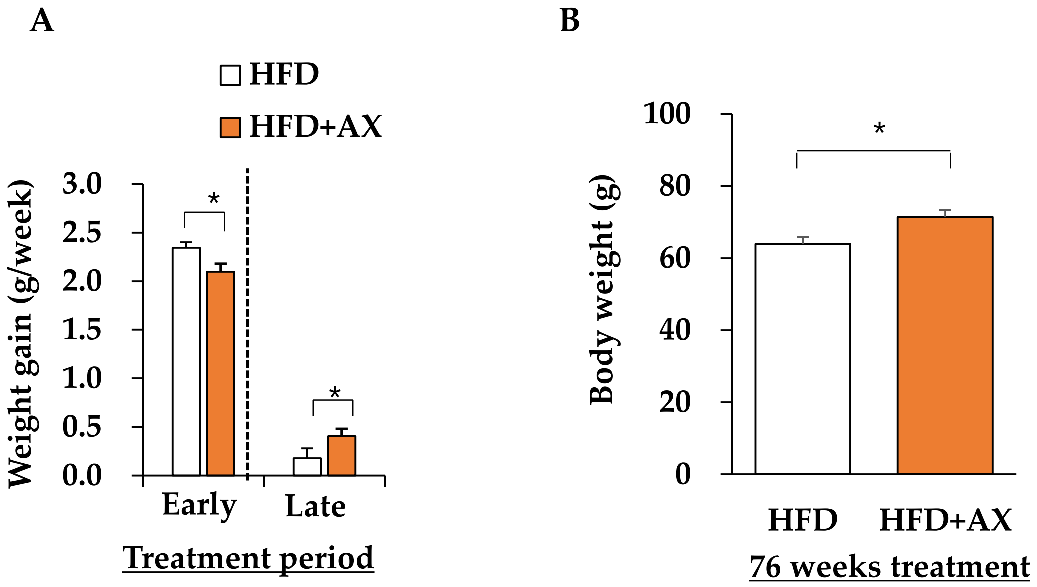

Figure S1. Administration of AX leads to characteristic weight changes against high-fat diet (HFD)-induced obesity in male C57BL/6J mice.
(A) Weight gain stratified by early (0-8 weeks) and late (16-24 weeks) weight change in Ref. [10] (HFD group, *n* = 10. HFD+AX group, *n* = 10). Six-week-old male mice were fed HFD for 24 weeks. (B) Body weights of C57BL/6J mice fed HFD alone (HFD group, *n* = 5) or HFD supplemented with AX (HFD+AX group, *n* = 5). Six-week-old male mice were fed HFD for 76 weeks. All values are presented as the means ± S.E.M. *$p < 0.05$, (HFD vs. HFD+AX). Statistical tests were performed as follows: (A,B) Student's *t* test.

Supplementary Figure S2

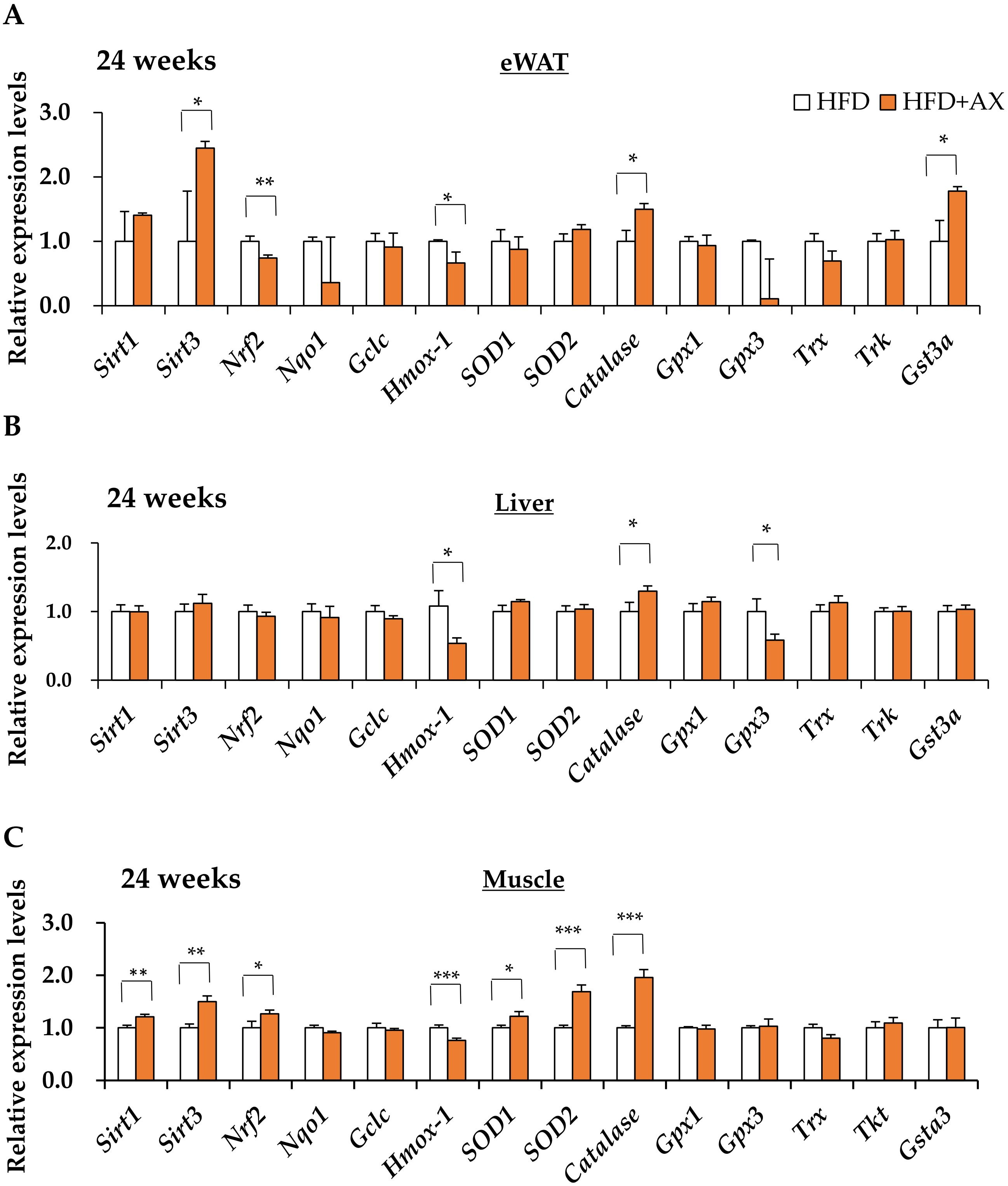

Figure S2. AX partially regulates oxidative stress response gene expressions in adipose tissue of HFD induced obese male C57BL/6J mice. Gene expressions in the eWAT (A) , liver (B), and gastrocnemius skeletal muscle (C) relative to that of *Tf2b* (A,B) or β-*actin* (C) expression, including of transcription factors related antioxidant enzyme and antioxidant enzymes in AX-treated HFD mice compared with control HFD mice. (n = 6 per group). All values are presented as the means ± S.E.M.*p < 0.05, **p < 0.01, ***p < 0.001 (HFD vs. HFD+AX). Statistical analysis was performed using Student's t-test.

Supplementary Figure S3

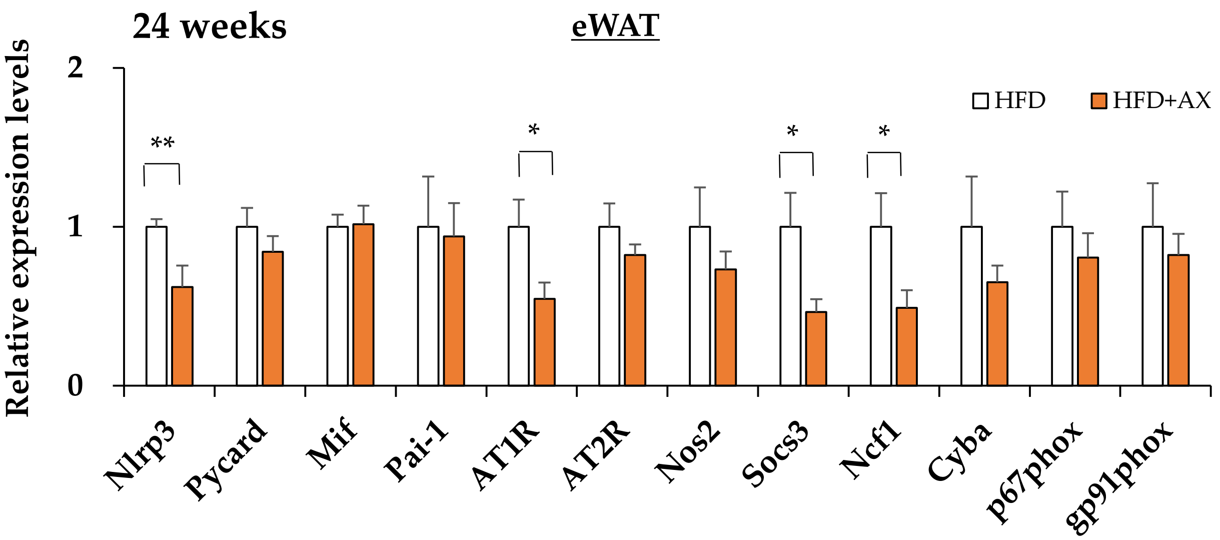

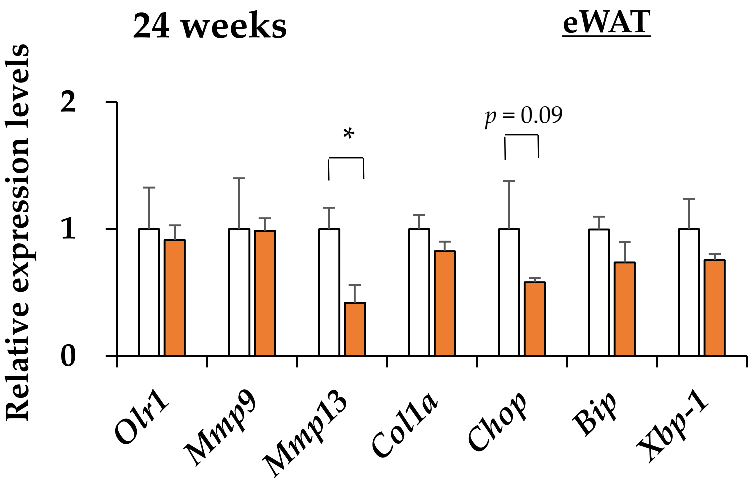

Figure S3. AX administration decreased the expression of diverse inflammatory markers in the eWAT compared to HFD treated control mice for 24 weeks. (n = 5-6 per group). All values are presented as the means ± S.E.M.*$p < 0.05$, **$p < 0.01$ (HFD vs. HFD+AX). Statistical analysis was performed using Student's t-test.

Supplementary Figure S4

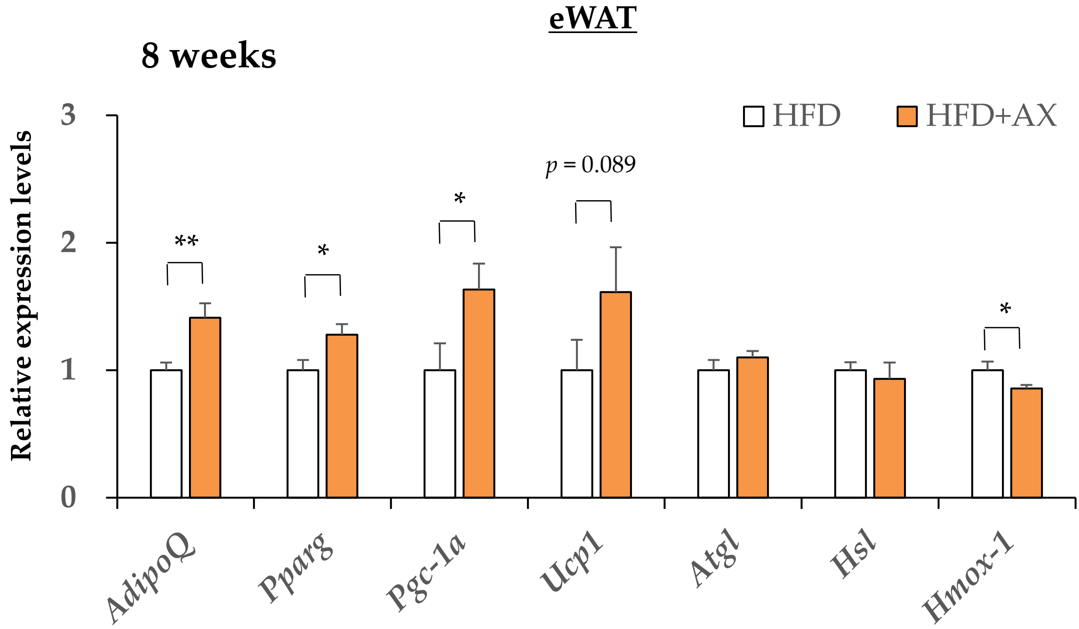

Figure S4. In 8-week high-fat diet-treated mice, AX treatment upregulated metabolism-related genes and downregulated oxidative stress marker genes in eWAT compared to the control HFD group.(n = 6 per group). All values are presented as the means ± S.E.M.*p < 0.05, .*p < 0.01 (HFD vs. HFD+AX). Statistical analysis was performed using Student's t-test.

Supplementary Figure S5

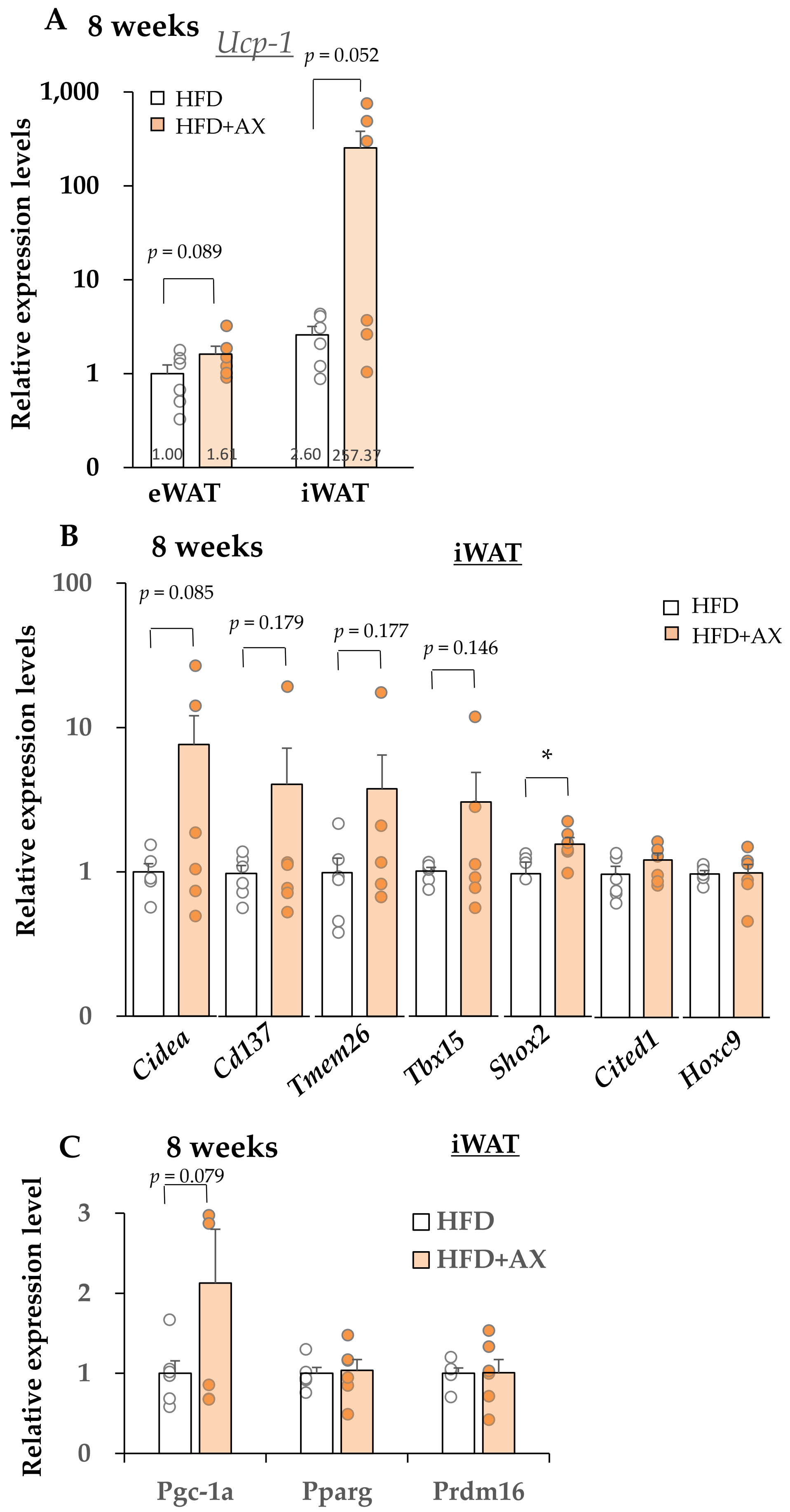

Figure S5. AX treatment transformed part of the inguinal subcutaneous adipose tissues (iWAT) in HFD mice to beige or bright. In AX-treated mice, the adipocytes in the subcutaneous adipose tissue were partially beige or bright, with high expression of uncoupling protein 1 (UCP-1). Gene expression levels of *Ucp-1* gene in iWAT and eWAT of obese mice fed HFD for 8 weeks (A). Gene expression levels of beige or bright adipocyte markers (B), and their associated transcription factors in iWAT (C). (n = 6 per group). All values are presented as the means ± S.E.M.*p < 0.05 (HFD vs. HFD+AX). Statistical analysis was performed using Student's t-test.

Supplementary Figure S6

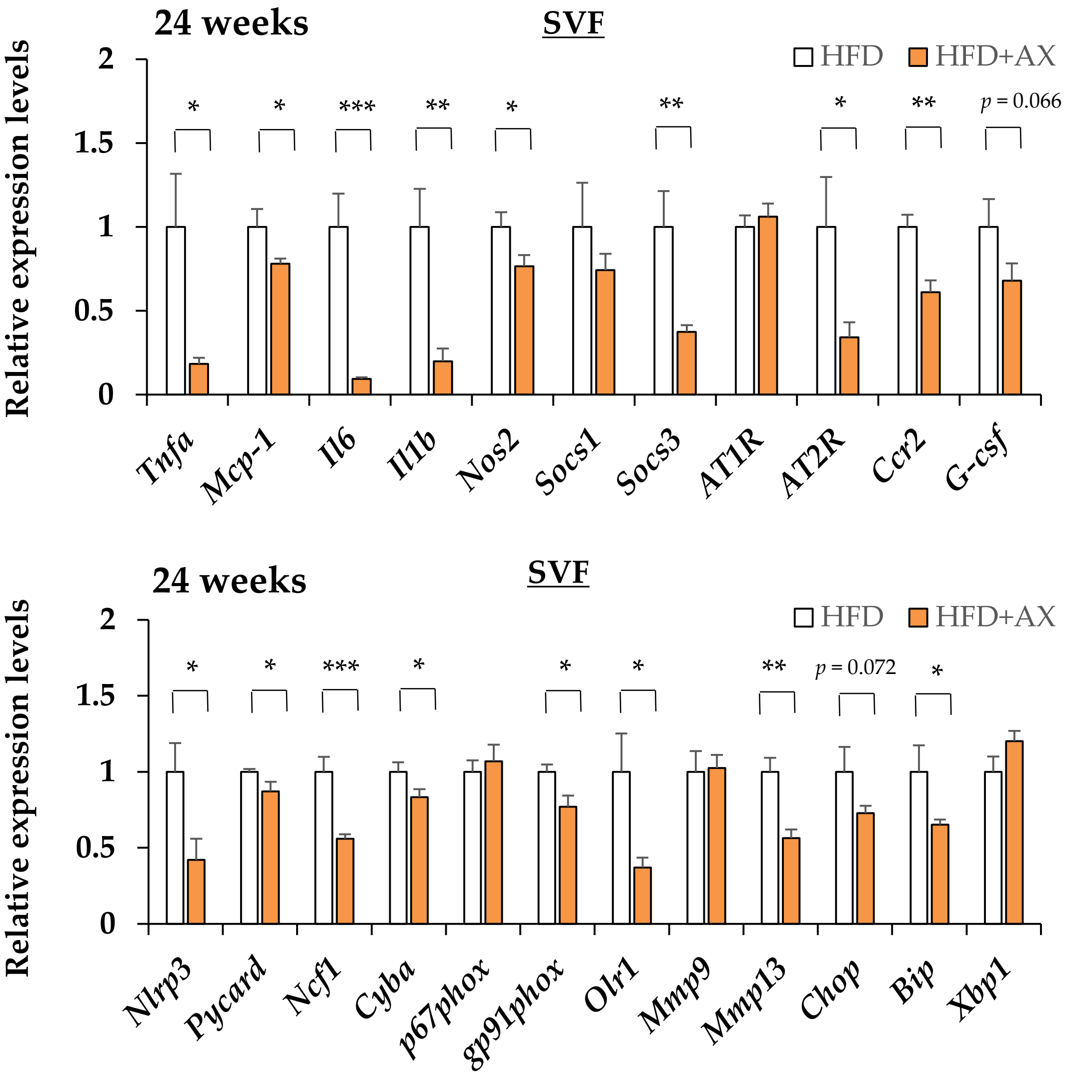

Figure S6. AX administration decreased the expression of diverse inflammatory markers in the SVF from eWAT compared to HFD treated control mice for 24 weeks. (n = 6 per group). All values are presented as the means ± S.E.M.*$p < 0.05$.**$p < 0.01$.***$p < 0.01$ (HFD vs. HFD+AX). Statistical analysis was performed using Student's t-test.

Supplementary Figure S7

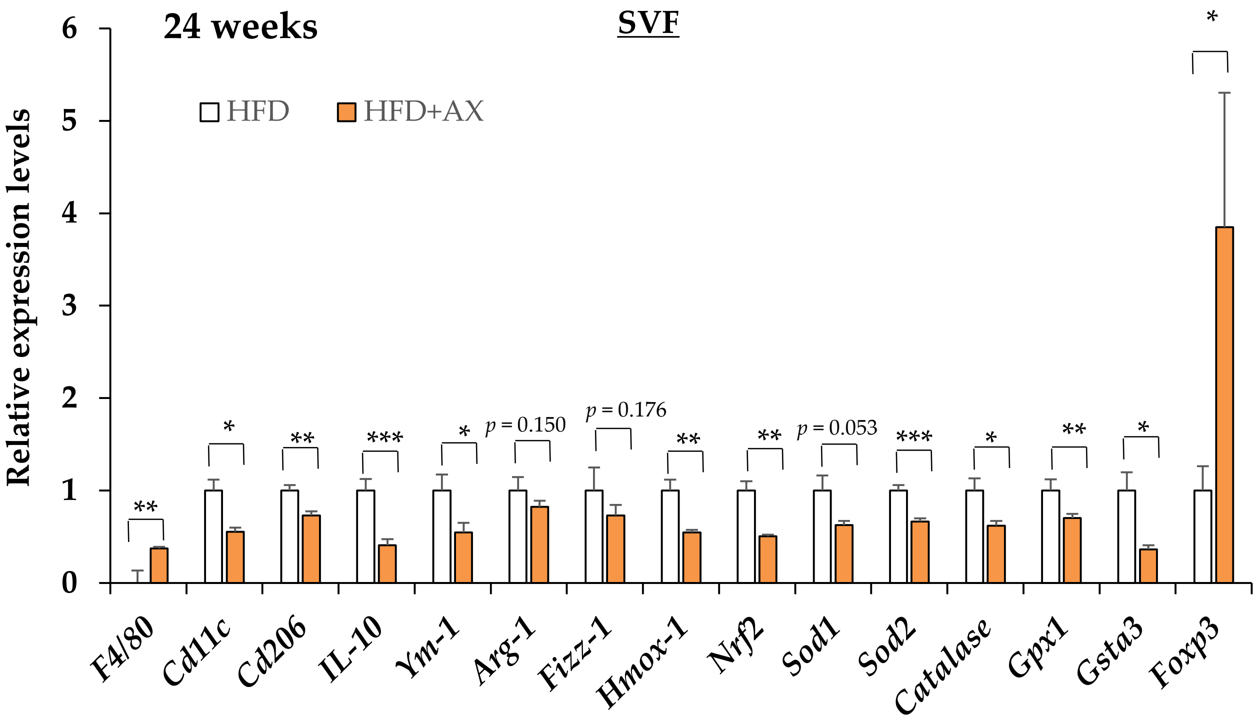

Figure S7. AX administration also decreased the expression of anti-inflammatory markers and oxidative stress markers in the SVF from eWAT compared to HFD-treated control mice for 24 weeks. (n = 6 per group). All values are presented as the means ± S.E.M. *$p < 0.05$.**$p < 0.01$.***$p < 0.001$ (HFD vs. HFD+AX). Statistical analysis was performed using Student's t-test.

Supplementary Figure S8

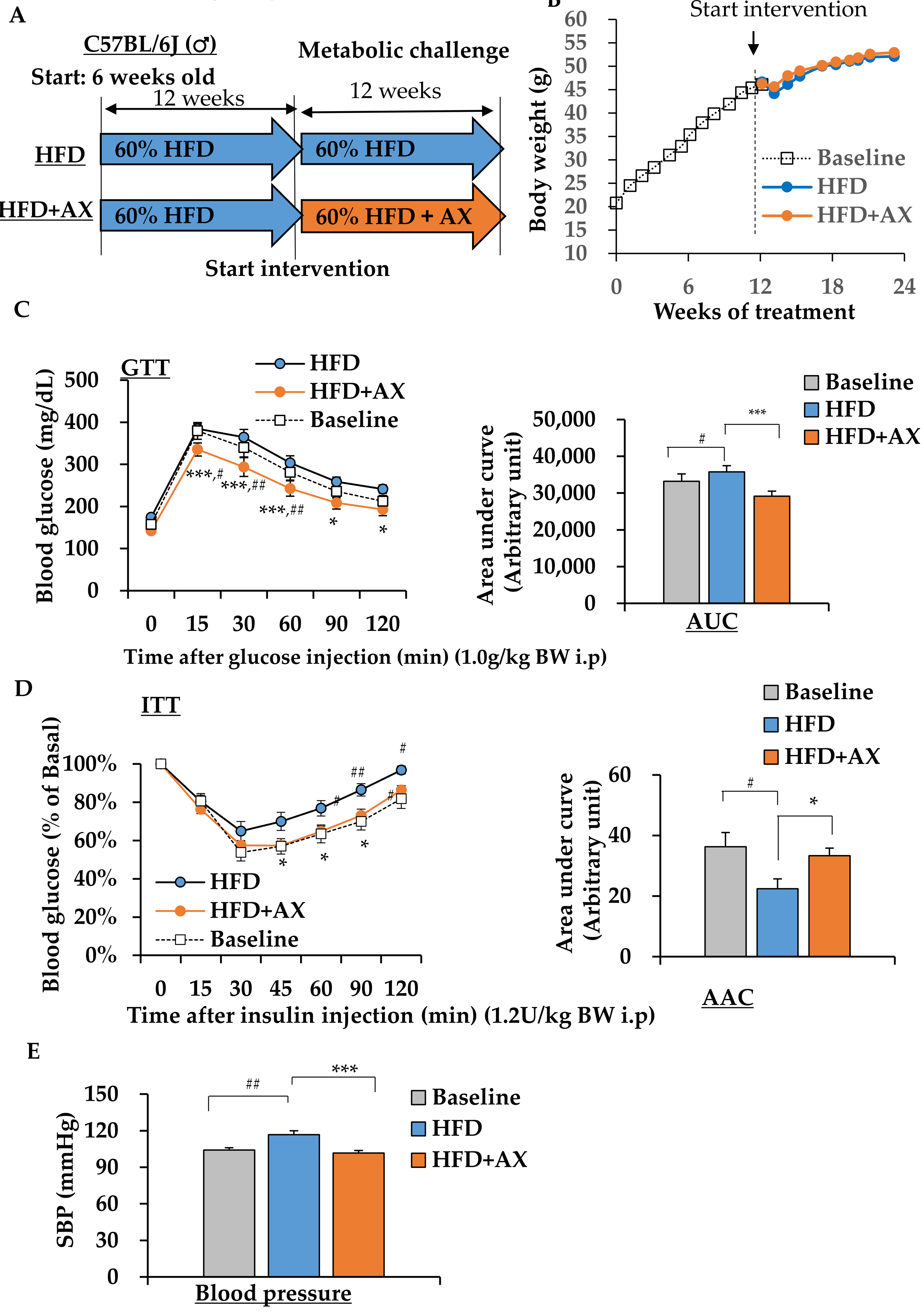

Figure S8. Intervention with AX in mice after the completion of pathological obesity improved insulin resistance and glucose intolerance. Mice were subjected to each intervention for 12 weeks after the 12 weeks of HFD treatment (A). Changes in body weight of mice in each group (B). Before and after 12 weeks of intervention, each group was measured for glucose tolerance (C), insulin sensitivity (D), and blood pressure (E). (*n* = 6-12 per group). All values are represented as means ± S.E.M. $^{*}p < 0.05$, $^{**}p < 0.01$, $^{***}p < 0.001$ (HFD vs. HFD+AX). $^{\#}p < 0.05$, $^{\#\#}p < 0.01$, $^{\#\#\#}p < 0.001$ (Baseline vs. each group). Statistical tests were performed as follows: (B) Student's *t*-test (C; IP-GTT, D; IP-ITT) two-way repeated-measures ANOVA, a post-hoc Tukey-Kramer for each point, and one-way ANOVA, a post-hoc Tukey-Kramer for AUC (C) or AAC (D), one-way ANOVA, a post-hoc Tukey-Kramer (E).
